# Three-Dimensional Evaluation of Accessory Canals in Primary Maxillary Molars Using Micro-CT

**DOI:** 10.1590/0103-644020256744

**Published:** 2026-06-26

**Authors:** Alice Moura Martins, Juliana de Medeiros Matos, Carolina Oliveira de Lima, Ana Flávia Almeida Barbosa, Emmanuel João Nogueira Leal da Silva, Tatiana Kelly da Silva Fidalgo

**Affiliations:** 1 Department of Preventive and Community Dentistry, School of Dentistry, State University of Rio de Janeiro (UERJ), Rio de Janeiro, RJ, Brazil; 2 Department of Dentistry, Federal University of Juiz de Fora, Governador Valadares, MG, Brazil; 3 Department of Endodontics, School of Dentistry, State University of Rio de Janeiro (UERJ), Rio de Janeiro, RJ, Brazil; 4 Department of Endodontics, School of Dentistry, Grande Rio University (UNIGRANRIO), Rio de Janeiro, RJ, Brazil

**Keywords:** Primary tooth, Molar, Endodontics, X-ray microtomography

## Abstract

The objective of the present study was to characterize the internal anatomy of primary maxillary molars by evaluating the presence and distribution of accessory canals using micro-computed tomography (micro-CT). Seventeen primary maxillary molars from donors aged 2 to 8 years were scanned using micro-CT and reconstructed using NRecon software. Only teeth with at least two-thirds of the root structure preserved were included. Image analysis was performed with ImageJ, supplemented by CTan and CTvol software. Accessory canals were identified and counted at each root and categorized by root third. Statistical analyses, including Kruskal-Wallis and Mann-Whitney tests, were applied (p<0.05). A total of 47 roots were analyzed, with 68.08% exhibiting at least one accessory canal, totaling 96 canals. The apical third showed a higher number of accessory canals compared to cervical (p=0.01), with a mean of 0.87±1.05, accounting for 42.71% of all accessory canals identified. The middle third had a mean of 0.80±1.42, representing 39.58% of the total, while the cervical third exhibited the lowest mean (0.36±0.84), corresponding to 17.71% of the overall canals. Notably, 57.44% of the apical thirds had at least one accessory canal. Also, considering the presence of at least one accessory canal, the apical third also presented a higher prevalence (p<0.01). Accessory canals are highly prevalent in the apical third, highlighting the complexity of their internal anatomy and the importance of thorough endodontic management

## Resumo

O objetivo do presente estudo foi caracterizar a anatomia interna de molares maxilares decíduos avaliando a presença e distribuição de canais acessórios usando microtomografia computadorizada (micro-CT). Dezessete molares decíduos superiores de doadores com idades entre 2 e 8 anos foram escaneados por micro-CT e reconstruídos utilizando o software NRecon. Os dentes deveriam apresentar pelo menos dois terços da raiz preservados. A análise de imagens foi realizada com ImageJ, suplementado pelos softwares CTan e CTvol. Os canais acessórios foram identificados e contados em cada raiz e categorizados por terço radicular. Análises estatísticas, incluindo os testes de Kruskal-Wallis e Mann-Whitney, foram aplicadas (p < 0,05). Um total de 47 raízes foram analisadas, com 68,08% exibindo pelo menos um canal acessório, totalizando 96 canais. O terço apical apresentou maior número de canais acessórios em comparação ao cervical (p = 0,01), com média de 0,87 ± 1,05, representando 42,71% de todos os canais acessórios identificados. O terço médio apresentou média de 0,80±1,42, representando 39,58% do total, enquanto o terço cervical apresentou a menor média (0,36±0,84), correspondendo a 17,71% do total de canais. Notavelmente, 57,44% dos terços apicais apresentaram pelo menos um canal acessório. Considerando também a presença de pelo menos um canal acessório, o terço apical também apresentou maior prevalência (p<0,01). Canais acessórios são altamente prevalentes no terço apical, destacando a complexidade de sua anatomia interna e a importância de um manejo endodôntico completo.

**Figure:**
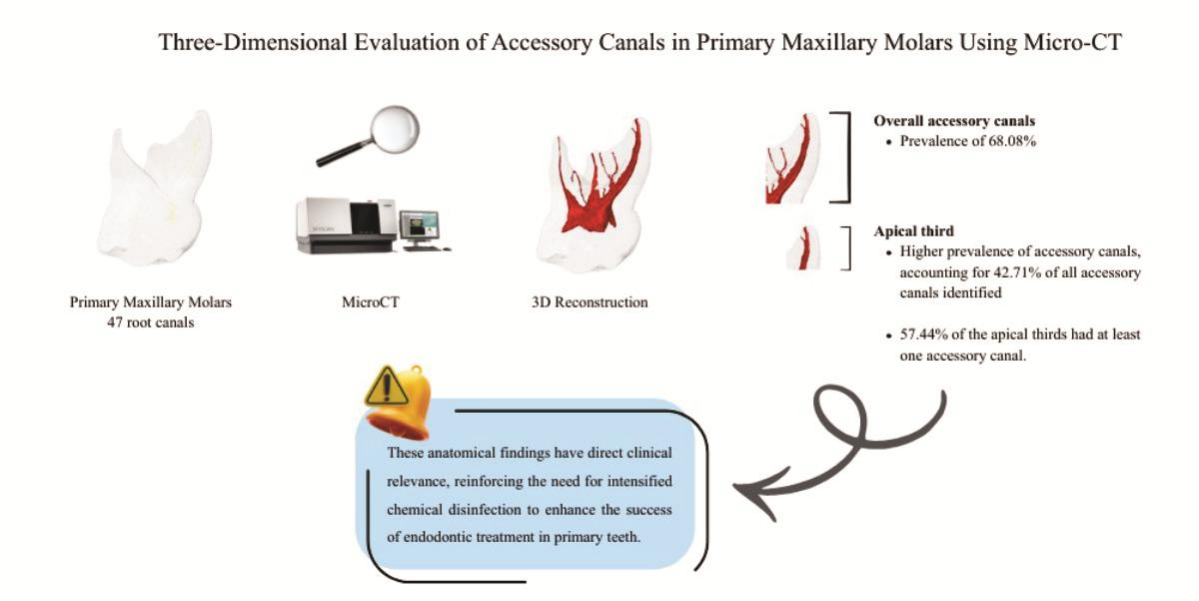

## Introduction

Pulpectomy in primary teeth is indicated in cases of carious or traumatic pulp exposures that result in necrosis or irreversible pulpitis ^1^. The effective reduction of bacteria through chemical-mechanical preparation and filling of the root canal system is essential for the success of the treatment^2^ ^,^ ^3^. However, achieving this goal is challenging due to the complex and variable anatomy of their root canal systems, which often contain branches, irregularities, and isthmuses that are difficult to detect and debride, thus compromising microbial reduction ^(^ ^4^ ^,^ ^5^ ^,^ ^6^. Therefore, addressing these hard-to-reach areas is essential for achieving predictable clinical success ^(^ ^7^ ^,^ ^8^ ^,^ ^9^.

The anatomical features of primary teeth have been extensively investigated using various methodologies ^(^ ^10^ ^,^ ^11^ ^,^ ^12^. Among these, micro-computed tomography (micro-CT) has emerged as a highly accurate, non-destructive imaging technique for evaluating internal morphology. It is now widely adopted in dental research, enabling quantitative and qualitative analyses, providing detailed information on parameters such as root canal curvature, diameter, surface area, and volume ^(^ ^12^ ^,^ ^13^ ^,^ ^14^. Due to its high resolution and ability to preserve sample integrity, micro-CT is currently considered the gold standard for assessing the internal anatomy of teeth ^(^ ^12^.

Accessory canals in primary molars represent relevant anatomical pathways that may influence disease progression and treatment outcomes. These ramifications can serve as additional routes for bacterial penetration and dissemination during pulp necrosis, allowing contamination beyond the central canal system^15^. Consequently, their presence and distribution may contribute to persistent infection and hinder adequate disinfection during endodontic procedures, thereby compromising outcomes ^(^ ^16^. Understanding their prevalence and morphology is therefore clinically relevant, as it helps anticipate anatomical complexity and supports more precise planning of cleaning, shaping, and obturation strategies in primary teeth ^(^ ^17^. In primary teeth, physiological root resorption typically begins approximately 3 to 4 years before the expected exfoliation age ^(^ ^18^ ^,^ ^19^. Clinically, the apical region may remain exposed to bacterial penetration during this period, potentially serving as a route for microbial dissemination and contributing to endodontic failure if not adequately disinfected.

Despite the existence of numerous studies examining root canal morphology and anatomical variations in primary teeth, including their ramifications ^(^ ^5^ ^,^ ^11^ ^,^ ^20^, there is still a lack of data specifically addressing the quantitative assessment of accessory canals in primary maxillary molars ^(^ ^21^. This gap in the literature is particularly relevant given the greater susceptibility of molars to carious lesions and the high global prevalence of dental caries in primary dentition, which is 46.2% ^(^ ^22^. Furthermore, the preservation of primary teeth through effective endodontic treatment plays a crucial role in maintaining arch space and positively influencing oral health-related quality of life ^(^ ^23^ ^,^ ^24^. A better understanding of the presence and distribution of accessory canals in primary molars may enhance the quality of treatment outcomes. Therefore, the aim of this study was to characterize the internal anatomy of primary maxillary molars, with a focus on identifying and analyzing accessory canals using micro-computed tomography.

## Materials and methods

### Sample Size Calculation

As there are no previous studies in the literature specifically evaluating the presence of accessory canals in primary maxillary molars, the sample size was calculated using an estimated proportion of 63.6%, as reported by a Brazilian study on maxillary primary molars, representing a high-variability condition for sample size estimation ^(^ ^25^. ^(^ ^25^A confidence level of 95% and a margin of error of 25% were adopted for this exploratory investigation. Using the standard formula for estimating proportions in descriptive studies:

$$n=\frac{Z^{2}\;x\;P(1-P)}{e^{2}}$$

where Z = 1.96 (for 95% confidence), P = 0.5 (estimated proportion), and e = 0.25 (margin of error), the resulting minimum sample size was approximately 14 teeth. Considering an expected 20% loss, 17 teeth were included in the present study.

### Sample Selection and Micro-CT analysis

This study was approved by the Local Research Ethics Committee (protocol number 4.568.832). The samples, composed of primary teeth, were collected from the Human Tooth Biobank of the School of Dentistry at UERJ (BDH) and the Human Tooth Biobank of FOUSP. The primary reason for extraction was extensive carious destruction that rendered the tooth unrestorable. Informed consent was obtained from the donors or their legal guardians. Physiological root resorption of primary molars typically begins approximately 3 to 4 years before exfoliation; therefore, we included teeth from donors from 2 to 8 years of age ^18^ ^,^ ^19^. A total of 17 extracted primary maxillary molars were selected; most of them (n = 14) were first molars, and the other (n = 3) were second molars, comprising a total of 51 roots. For inclusion criteria, only roots that presented at least two-thirds of extension were selected. Therefore, roots exhibiting more than one-third of physiological resorption were excluded, as such resorption compromises the assessment of anatomical features, particularly in the middle and apical thirds. Consequently, four roots were excluded, resulting in a final sample of 47 roots analyzed. In cases where teeth exhibited partial resorption, the position of accessory canals (cervical, middle, and apical) was estimated based on the natural taper and convergence of the roots, and by comparing the affected roots with the unaffected roots from the same tooth.

During the micro-CT assessment, the evaluator was blinded and conducted the analysis using numerical codes assigned to the teeth. The teeth were scanned using a SkyScan 1174 microtomograph (Bruker-microCT, Kontich, Belgium), at 50kV, 800mA, 0.8 rotation step, 180° rotation around the vertical axis, 4500ms exposure time, and 0.5mm aluminum filter. The images were then reconstructed using NRecon software v. 1.6.8.0 (Bruker micro-CT, SkySCAN, Kontich, Belgium) using 20% beam hardening correction, ring artifact correction of 10, and smoothing of 2, resulting in the acquisition of 400 to 700 axial cross sections per sample. After the reconstruction of the images, 3D images of the roots and root canal systems were created by CTAn v.1.14.4 and CTvol v.1.6.6.0 (Bruker, Kontich, Belgium) software.

An analysis of the internal anatomy was conducted using ImageJ software v. 1.50d (National Institutes of Health, Maryland, USA). A complementary analysis of the 3D images was also performed using CTvol v.1.6.6.0 software.

Each root was individually examined for the presence, number, and location of accessory canals. The number of accessory canals was recorded according to root type (mesiobuccal, distobuccal, or palatal) and root third (cervical, middle, or apical). For the purposes of this study, all canal ramifications branching from the central canal, regardless of their diameter or trajectory, were classified as accessory canals ^12^ ^,^ ^16^.

### Intraclass Correlation Coefficient (ICC)

To assess intra-examiner reliability, six permanent maxillary molars were selected. The evaluator recorded the number of accessory canals in each root, classifying them by root type (mesiobuccal, distobuccal, and palatal) and by root third (cervical, middle, and apical), using ImageJ software v.1.50d (National Institutes of Health, Maryland, USA). The evaluation was repeated after a 15-day interval under the same conditions. Data were analyzed using SPSS software v.25.0 (IBM Corp., Armonk, New York, USA), and the intraclass correlation coefficient (ICC) was calculated.

### Statistical analysis

The data were entered into SPSS software v.25.0 (IBM Corp., Armonk, New York, USA). The analysis of accessory canals was performed by categorizing findings according to root type (mesiobuccal, distobuccal, and palatal) and root third (cervical, middle, and apical). Data normality was assessed using the Shapiro-Wilk test (p < 0.05). As the data did not follow a normal distribution, non-parametric tests were applied: the Kruskal-Wallis test for comparisons among multiple groups, and the Mann-Whitney U test for pairwise comparisons. The confidence level was set at 95%, and statistical significance was established at p < 0.05.

## Results

**Figure 1:**
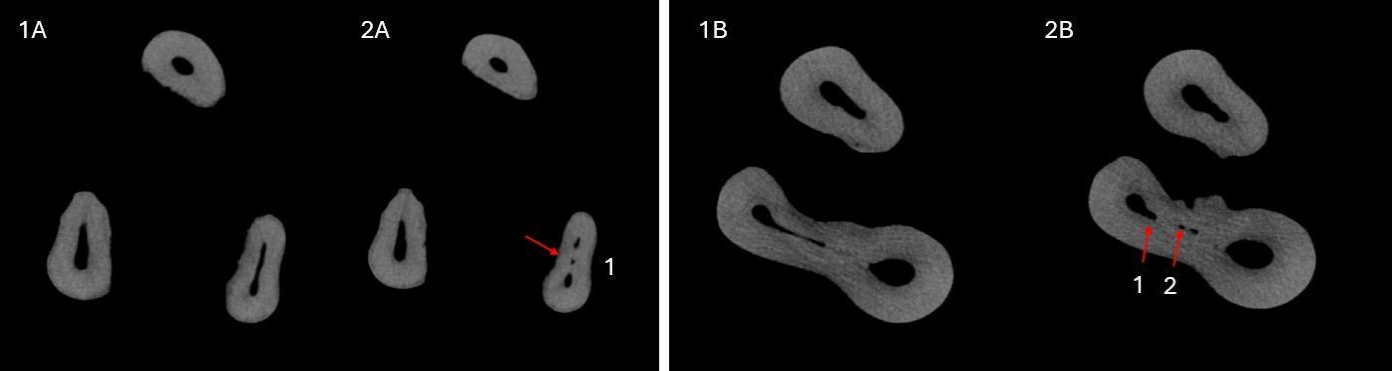
Axial sections of micro-computed tomography of upper primary first molars showing the root canal system of two different teeth from the sample. Images 1A and 2A correspond to different axial sections of the same sample, while images 1B and 2B correspond to different axial sections of another sample. Some accessory canals are indicated by red arrows. In A, the arrow demonstrates one accessory canal (1A and 2A); in B, the arrows indicate two accessory canals (1B and 2B).

**Figure 2:**
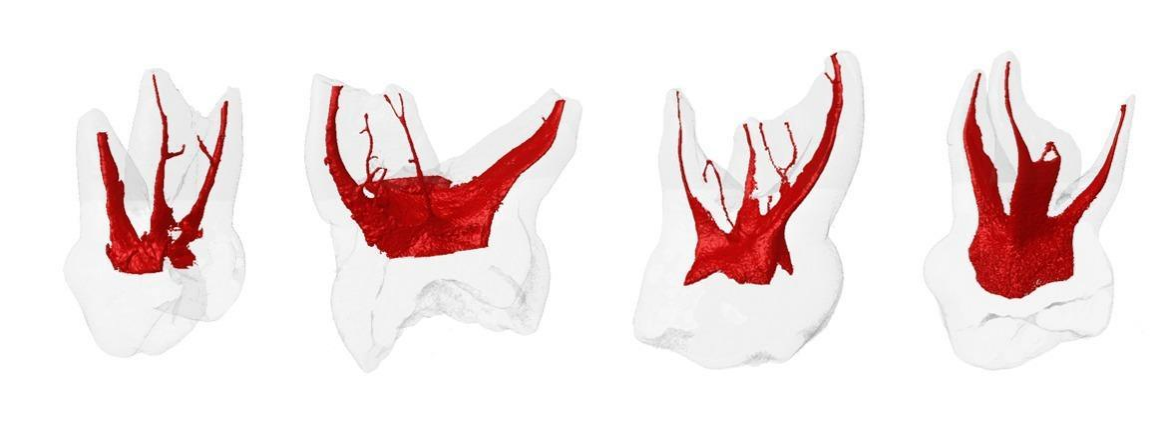
Three-dimensional representation of three different primary maxillary molars.

Figure 1 shows axial sections in the ImageJ software illustrating accessory canals in different roots, and Figure 2 exhibits 3D images of teeth from the sample in the CTvol software. The ICC value obtained was 0.99, indicating excellent intra-examiner agreement.

A total of 96 accessory canals were identified, with 68.08% of the roots presenting at least one accessory canal. Table 1 summarizes the distribution of accessory canals according to root type. There was no statistically significant difference in the overall number of accessory canals among the three root types (p = 0.30), nor in the presence of at least one accessory canal (p = 0.06). The palatal roots exhibited a mean of 2.37 ± 2.82 accessory canals, accounting for 39.58% of all accessory canals identified. The distobuccal roots had a mean of 2.00 ± 1.81 accessory canals (31.25%), while the mesiobuccal roots showed a mean of 1.75 ± 3.27 (29.19%). Despite not having the highest mean, 86.66% of the distobuccal roots contained at least one accessory canal, representing the highest frequency of occurrence among the three root types.

**Table 1:** Number of canals, mean, standard deviation and presence of at least 1 accessory canal per root.

Root	Overall Canal N (%)	Mean ± SD	Presence of at Least 1 Accessory Canal (%)
Mesiobuccal	28 (29.17)	1.75 ± 3.27	8 (50.00)
Distobuccal	30 (31.25)	2.00 ± 1.81	13 (86.66)
Palatal	38 (39.58)	2.37 ± 2.82	11 (68.75)

Note: Statistical differences are indicated with *; Kruskal-Wallis and Mann-Whitney test (p<0.05).

Legend: N: number of accessory canals found; SD: Standard Deviation.

Table 2 presents the distribution of accessory canals according to root thirds. The apical third showed a statistically higher number of accessory canals compared to the cervical third (p = 0.01), with a mean of 0.87 ± 1.05, accounting for 42.71% of all accessory canals identified. The middle third had a mean of 0.80 ± 1.42, representing 39.58% of the total, while the cervical third exhibited the lowest mean (0.36 ± 0.84), corresponding to 17.71% of the overall canals. Notably, 57.44% of the apical thirds had at least one accessory canal. Also, considering the presence of at least one accessory canal, the apical third also presented a higher prevalence (p < 0.01).

**Table 2:** Number of canals, mean, standard deviation and presence of at least 1 accessory canal per third of the root canal system.

Third of Root Canals	Overall Canal N (%)	Mean ± SD	Presence of at Least 1 Accessory Canal (%)
Cervical	17 (17.71)*	0.36 ± 0.84	12 (25.53)*
Middle	38 (39.58)	0.80 ± 1.42	19 (40.42)
Apical	41 (42.71)*	0.87 ± 1.05	27 (57.44)*

Note: Statistical differences are indicated with *; Kruskal-Wallis and Mann-Whitney test (p<0.05).

Legend: N: number of accessory canals found; SD: Standard Deviation.

Table 3 displays the distribution of accessory canals according to both root type and root third. When correlating these two parameters, no statistically significant difference was found (p = 0.153). The middle third of the palatal root exhibited the highest number of accessory canals, with a total of 17 canals. This was followed by the apical third of the palatal and distobuccal roots, each with 14 accessory canals. Notably, 80% of the apical thirds of the distobuccal roots presented at least one accessory canal, indicating a high frequency in this region. There was no difference considering first or second molars. From MV roots, 93.75% presented three-thirds of extension, from DV, 60.00%, and from P, 75.00%.

**Table 3.** Number of canals found, mean, standard deviation and presence of at least 1 accessory canal per available root and root third.

Overall	Cervical			Middle			Apical		
Root	Overall Canal N (%)	Mean ± SD	Presence of at Least 1 Accessory Canal (%)	Overall Canal N (%)	Mean ± SD	Presence of at Least 1 Accessory Canal (%)	Overall Canal N (%)	Mean ± SD	Presence of at Least 1 Accessory Canal (%)
MB	7 (7.29)	0.43 ± 1.26	3 (18.75)	8 (8.33)	0.50 ± 0.89	5 (31.25)	13 (13.54)	0.81 ± 1.32	7 (43.75)
DB	3 (3.13)	0.20 ± 0.41	3 (20.00)	13 (13.54)	0.86 ± 1.40	7 (46.66)	14 (14.58)	0.93 ± 0.59	12 (80.00)
P	7 (7.29)	0.43 ± 0.62	6 (37.50)	17 (17.71)	1.06 ± 1.84	7 (43.75)	14 (14.58)	0.87 ± 1.14	8 (50.00)
1st Molar									
MB	1 (1.56)	0.07 ± 0.28	1 (7.69)	2 (3.13)	0.15 ± 0.38	2 (15.38)	5 (7.81)	0.38 ± 0.65	4 (30.77)
DB	3 (4.69)	0.21 ± 0.43	3 (21.43)	12 (18.75)	0.85 ± 1.46	6 (42.86)	12 (18.75)	0.86 ± 0.53	11 (78.57)
P	4 (6.25)	0.31 ± 0.63	3 (23.08)	16 (25.00)	1.23 ± 2.00	6 (46.15)	9 (14.06)	0.69 ± 1.18	5 (38.46)
2nd Molar									
MB	6 (18.75)	2.00 ± 2.64	2 (66.67)	6 (18.75)	2.00 ± 1.00	3 (100.00)	8 (25.00)	2.67 ± 2.08	3 (100.00)
DB	0 (0.00)	0.00 ± 0.00	0 (0.00)	1 (3.13)	1.00 ± 0.00	1 (100.00)	2 (6.25)	2.00 ± 0.00	1 (100.00)
P	3 (9.38)	1.00 ± 0.00	3 (100.00)	1 (3.13)	0.33 ± 0.58	1 (33.33)	5 (15.63)	1.67 ± 0.58	3 (100.00)

Note:Statistical differences are indicated with *; Kruskal-Wallis and Mann-Whitney test (p<0.05).

Legend: N: number of accessory canals found; SD: Standard Deviation; MB: Mesiobuccal; DB: Distobuccal; P: Palatal.

## Discussion

A total of 68.08% of upper maxillary molars exhibited at least one accessory canal. The present study found a similar number of accessory canals in the different root types of primary maxillary molars. However, when considering the location within the root, the apical third exhibited a significantly higher number of accessory canals compared to the cervical third. These findings underscore the anatomical complexity of the root canal system in primary teeth, particularly within the apical region, and reinforce the potential challenge this poses for decontamination and obturation. In clinical terms, unrecognized accessory pathways in the apical third may act as additional routes for microbial persistence, which could adversely affect the success of endodontic treatment in primary teeth. Although there are studies in the literature addressing the internal anatomy and root canal morphology of primary teeth, much of this research has focused primarily on parameters such as the Vertucci classification, curvature, number, and shape of root canals, among other aspects ^5^ ^,^ ^26^ ^,^ ^27^. Studies investigating root canal ramifications in primary dentition have predominantly concentrated on the furcation area, examining the presence, location, and size of accessory canals specifically in this region ^20^ ^,^ ^28^, as well as their role in the pathogenesis of endodontic infections ^11^. Sharma et al. (2016) analyzed primary molars using scanning electron microscopy and reported the presence of accessory canals in the furcation region in 73,3% of the sample ^11^. Kumar (2009) analyzed 15 primary maxillary first molars and 15 second molars and found accessory canals in the pulpal floor in 60% and 53.3% of samples, respectively ^20^. Despite the relevance of these studies, direct comparisons with the present research are limited because they focus on accessory canals in a specific region of the root canal system, whereas the current study evaluated accessory canals throughout the entire root canal system, including the cervical, middle, and apical thirds. Moreover, the objectives differ, as this study emphasizes a comprehensive quantitative assessment using micro-CT technology, which offers high-resolution three-dimensional visualization.

Regarding morphological analyses of the root canal system of primary teeth that used micro-CT, Teixeira et al. (2023) investigated the lateral canals of maxillary molars ^21^. Their sample included maxillary molars, and the authors did not provide an overall lateral canal prevalence, but it was identified that 44.8% of lateral canals were located in the apical third. The present study identified 68.8% of accessory canals, 42.7% of these canals located in the apical third, indicating a consistent pattern regarding the preferential location of root canal ramifications. The difference in the total number of accessory canals detected between the two studies may be attributed to methodological differences. The literature shows that the prevalence of accessory canals in permanent teeth is 80.71%. The apical third was the most common location for accessory canals in the mesial and distal roots ^29^. This prevalence is higher than the present study, which found 68.08%, but varies with population and methods used, and corroborates the higher prevalence in the apical third.

The significant presence of accessory canals found in this study highlights the challenge of thoroughly cleaning and filling primary molars. Filling materials need to have good flow to seal these small, complex spaces, especially in the apical third, where accessory canals are more common ^30^. However, even when filled, accessory canals may not be thoroughly disinfected, which can compromise treatment success ^31^. Irrigation is essential because mechanical instrumentation often cannot reach these canals ^6^. It is important to keep the irrigation needle close to the working length to maximize cleaning, especially in the apical third ^32^ ^,^ ^33^. Advanced irrigation techniques, like negative pressure systems, laser and ultrasonic activation, may improve cleaning effectiveness, but more clinical research is needed to confirm their practical benefits in primary teeth compared to the conventional syringe and needle technique ^34^ ^,^ ^35^ ^,^ ^36^.

The present study has some limitations. The sample size was limited due to the difficulty in obtaining primary teeth without significant root resorption, which is common in this population. Because of the small sample, no distinction was made between first and second molars. In cases with root resorption, estimating the position of accessory canals relative to the root thirds was necessary, which reduces the precision of their exact location. To avoid bias caused by excluding heavily resorbed roots, accessory canals were counted per root rather than per tooth. Although all canal ramifications were recorded, no differentiation was made between accessory canal types; only their location was noted. Finally, the scarcity of comparable studies on the number and location of accessory canals in primary maxillary molars limited the possibility of thorough comparisons with existing literature.

Despite the inherent challenges associated with physiological root resorption in primary teeth, this study offers several important contributions to the understanding of their internal anatomy. Utilizing high-resolution micro-computed tomography, it provides detailed and comprehensive data on the presence and distribution of accessory canals in primary maxillary molars-a subject that remains underexplored in the literature. By mapping these complex anatomical features throughout the entire root canal system, rather than focusing solely on specific regions such as the furcation area, this research broadens the scope of current knowledge and offers a more holistic view of root canal morphology in primary teeth. Furthermore, the study’s methodological approach, which includes a careful selection of roots and rigorous analysis protocols, strengthens the reliability of the findings.

The identification of a higher prevalence of accessory canals in the apical third highlights clinically relevant information that can directly impact endodontic treatment strategies, such as irrigation and filling techniques tailored to address these complex canal networks. These positive aspects underscore the study’s value as a foundational reference for future investigations. Given the scarcity of similar high-quality micro-CT analyses in primary molars, especially maxillary molars, the findings set the stage for further research aimed at correlating anatomical complexities with treatment outcomes. Ultimately, expanding this knowledge will be essential to refining clinical protocols and improving the prognosis of endodontic therapy in pediatric dentistry.

## Conclusion

The analysis of primary maxillary molar roots in this study revealed a prevalence of 68.08%, a high prevalence of accessory canals, underscoring the complexity of their internal anatomy. The apical third exhibited the most significant number of accessory canals. These anatomical findings have direct clinical relevance, reinforcing the need for intensified chemical disinfection to enhance the success of endodontic treatment in primary teeth.

## Data Availability

The research data are available within the article.
